# Electron nematic effect induced by magnetic field in antiferroquadrupole phase of CeB_**6**_

**DOI:** 10.1038/s41598-017-17608-3

**Published:** 2017-12-12

**Authors:** S. V. Demishev, V. N. Krasnorussky, A. V. Bogach, V. V. Voronov, N. Yu. Shitsevalova, V. B. Filipov, V. V. Glushkov, N. E. Sluchanko

**Affiliations:** 1 Prokhorov General Physics Institute of RAS, Vavilov street, 38, 119991 Moscow, Russia; 2 National Research University Higher School of Economics, Myasnitskaya street, 20, 101000 Moscow, Russia; 3 Institute for Problems of Materials Science of NASU, 3, Krzhyzhanovsky Str., Kiev, 03680 Ukraine; 4 Moscow Institute of Physics and Technology, 9 Institutsky lane, Dolgoprudny, 141700 Moscow, Russia

## Abstract

Spatial anisotropy generated spontaneously in the translationally invariant metallic phase, i.e. electron nematic effect, addresses a great challenge for both experimentalists and theoreticians. An interesting option for the realization of the electron nematic phase is provided by the system with orbital ordering, as long as both orbitally ordered states and electron nematic phases possess broken spatial symmetry. Here we report the detailed study of the angular dependences of the magnetoresistance in the orbitally ordered antiferroquadrupole (AFQ) phase of CeB_6_. Our data allowed revealing the electron nematic effect, which develops when magnetic field exceeds a critical value of 0.3–0.5T. As a result, new transition inside the AFQ phase corresponding to the change of the symmetry of magnetic scattering on spin fluctuations in CeB_6_ is discovered.

## Introduction

In the past decade, investigating of spatial anisotropy that is generated spontaneously in the translationally invariant metallic phase, i.e. electron nematic or spin nematic effect, has addressed a great challenge for both experimentalists and theoreticians^1–4^. Indeed, numerous experimental objects like ultra clean quantum Hall systems, ruthenates, high-T_c_ superconductors^1^ and iron-based superconductors^2–4^ demonstrate unexpected anisotropy in their electronic properties, forbidden by high symmetry of the studied systems. As pointed out in ref.^1^, it looks amazing how point-like electrons demonstrate properties similar to those of liquid crystals, where rod-shaped molecules act as basic blocks to form a nematic phase^5^. Up to now, several theoretical mechanisms were proposed to explain electron nematic effect including Pomeranchuk instability of Fermi liquid or melting of a stripe phase^1,2,4^. Another intriguing option for the realization of an electron nematic phase is provided by the crystal with orbital ordering, as long as both orbitally ordered states and electron nematic phases may be considered as systems with broken spatial symmetry^1,4^. In the quantum spin systems, nematic phases correspond to the case when the spin-rotational symmetry is broken but no magnetic order is formed^6^. This specific situation may occur in the phase with quadrupolar order, where the spin fluctuation magnitude varies along different crystallographic directions^6^, so that anisotropic spin fluctuations “mimic” molecules in the liquid crystal nematic phase.

As stressed in ref.^6^, observation of nematic effects in the quadrupolar or orbital phases is challenging. In thermodynamic studies, they behave as antiferromagnets^6^ and, consequently, the choice of proper experimental object is of crucial importance. A possible candidate for search of the nematic effects related to orbital order is cerium hexaboride, CeB_6_. This strongly correlated metal with highly symmetric cubic lattice is believed to represent an archetypal dense Kondo system with orbital ordering in the antiferroquadrupole (AFQ) phase at low temperatures *T* < *T*_*Q*_^7^. The choice to study nematic effects in CeB_6_ is also inspired by the recent study of electron spin resonance (ESR)^8^. In this material, ESR may be detected in the AFQ phase and is missing in the paramagnetic (P) phase, where orbital ordering is destroyed^8^. According to the modern point of view, the ESR line width in the Kondo systems is controlled by spin fluctuations^9^. Experiment shows that the observed ESR line width in CeB_6_ is strongly anisotropic and its angular dependence demonstrates a good correlation with the angular dependence of the magnetoresistance in the resonance field^8^. The established link between spin fluctuations anisotropy and resistivity of the sample is very important because studying of the resistivity anisotropy plays a key role in experimental establishing of electron nematic effect^2–4^.

It is worth noting that the analogy with liquid crystals is not confined to the case of nematic phases. For example, some phases with an intermediate magnetic order existing in the spiral magnets may be treated as replicas of blue fog phases in cholesteric liquid crystals^10,11^. However, there is an essential difference in construction of the order parameter for liquid crystals and electron/spin nematic phases. The order parameter in nematic liquid crystals may be introduced via angular distribution function *f*(*θ*)^5^, which is given by a sum of Legendre polynomials *P*_*n*_(*x*):

1 $$f(\theta )=\sum _{n=0}^{n=\infty }{a}_{n}{P}_{n}(\cos \,\theta ).$$

Here the angle *θ* is measured from the axis of nematic ordering (director)^5^. If the director is non-polar, the odd coefficients *a*_*n*_ (*n* = 1, 3, 5…) are equal to zero so that equation (1) acquires the form

2 $$f(\theta )=1+{a}_{2}{P}_{2}(\cos \,\theta )+{a}_{4}{P}_{4}(\cos \,\theta )+\mathrm{...},$$

where the coefficient *a*_2_ plays the role of an order parameter, which is zero in the isotropic phase and non-zero in the nematic phase^5^.

In the case of electron nematic phases, the order parameter is defined typically by relevant resistivity values (ρ_*xx*_ − ρ_*yy*_)/(ρ_*xx*_ + ρ_*yy*_), where *x* and *y* axes are some principal directions in the electron nematic phase^1–4^, e.g., corresponding to various magnitude of spin fluctuations^6^. The angular dependences of the resistivity in the electron nematic phase were measured in some works^12–14^, but to our best knowledge, the analysis of the *f*(*θ*) within equations (1–2) has never been performed. This fact constitutes a certain “gap” in analogy between strongly correlated electron systems and liquid crystals. It is worth noting, that the applicability of equation (1) to electron nematic effect is not trivial, because these phenomena develop in an anisotropic crystal medium, whereas the case of nematic liquid crystal corresponds to free rotation of a rod-shaped molecules in a solvent. Here we show that the comprehensive study of the angular dependences of magnetoresistance in the orbitally ordered AFQ phase of CeB_6_ is very useful to reveal the electron nematic effect, which develops in magnetic field *B* exceeding some critical value ~0.3–0.5 T. As a result, new transition inside the AFQ phase corresponding to the change of the symmetry of magnetic scattering on spin fluctuations is discovered.

**Figure 1:**
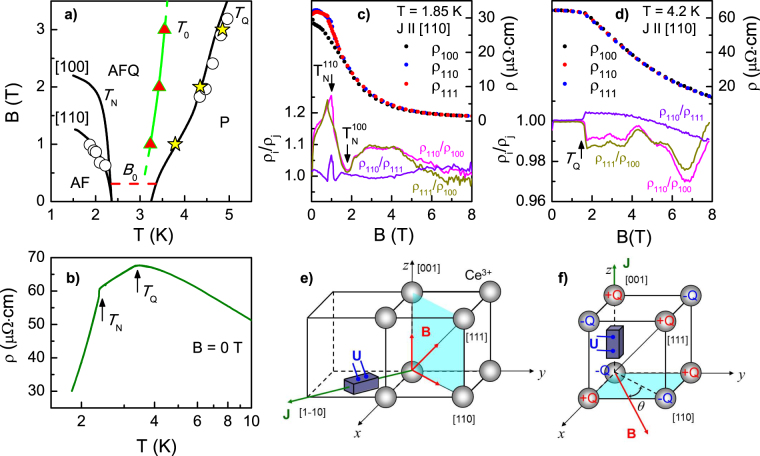
(**a**) Magnetic phase diagram of CeB_6_. Black solid lines correspond to the transitions into AFQ (*T*_*Q*_) and AF (*T*_*N*_) phases as detected by resistivity measurements^6^; open circles denote the results of microwave measurements in a magnetic field applied along [110] crystallographic direction^7^. Stars and triangles mark characteristic temperatures related to electron nematic effect in CeB_6_ (see text); *B*_0_ corresponds to expected threshold field (see text for details). (**b**) The temperature dependence of resistivity in zero magnetic field; *T*_*Q*_ and *T*_*N*_ are shown by arrows. (**c,d**) Magnetoresistance of CeB_6_ single crystal at 1.85 K and 4.2 K for different directions of magnetic field **B**||[001], **B**||[111] and **B**||[110] (excitation dc current **J**||[1–10]). The experimental data are shown by solid circles, the subscripts correspond to the field direction. Lines present the ratios ρ_i_/ρ_j_ (i, j = {100;110;111}). (**e,f**) The experimental layouts for measuring of the resistivity anisotropy in a magnetic field. The Ce^3+^ ions with the Γ_8_ ground state are located at the vertices of the cubic unit cell. In the panel f the orbitals corresponding to different quadrupolar moments are marked as +Q and −Q.

Before discussing of experimental layout and data, let us revisit briefly the *B-T* magnetic phase diagram of CeB_6_^7^ shown in Fig. 1a. The phase boundaries *T*_*Q*_(*B*) and *T*_*N*_(*B*) correspond to ordering of 4f orbitals and localized magnetic moments (LMMs) of Ce^3+^ ions, respectively. In the range *T* > *T*_*Q*_(*B*) the paramagnetic (P) phase is stable and both spins and orbitals are disordered. The orbital ordering of 4f^1^ states of Ce^3+^ at *T*_*Q*_(*B*) may be described as a formation of 3D chess board structure constructed of two sublattices containing positive and negative quadrupolar moments +Q and −Q originating from the Γ_8_ ground state wave functions^7,15^. As long as the external magnetic field induces a magnetic moment on the Γ_8_ states, the orbital ordering may be detected in neutron scattering experiment, where the field-induced staggered magnetization is proved to result in the antiferromagnetic (AF) reflex with the **k** = [1/2, 1/2, 1/2] wave vector corresponding to the doubling of the lattice period^7,15^. For that reason, the orbitally ordered state of CeB_6_ at *T* < *T*_*Q*_(*B*) is referred as antiferroquadrupole (AFQ) phase. In the range *T* < *T*_*Q*_(*B*) spins of Ce^3+^ ions remain disordered, until Néel transition temperature *T*_*N*_(*B*) is reached. Below *T*_*N*_(*B*) an extra doubling of the period of magnetic structure occurs and complicated AF phase with spin structure modulated by the wave vectors **k**_1_ = [1/4, 1/4, 1/2] and **k**_2_ = [1/4, −1/4, 1/2] develops^7,15–17^. Experiments show that *T*_*Q*_(*B*) increases in a magnetic field and this phase boundary is isotropic, whereas *T*_*N*_(*B*) depends on crystallographic direction and AF state may be suppressed by applying moderate field *B*~1-2 T (Fig. 1a).

The features originated from the orbital/magnetic ordering in CeB_6_ are clearly resolved in our resistivity data (Fig. 1b–d). For *B* = 0 the characteristic temperatures *T*_*Q*_ and *T*_*N*_ are detected as the kinks on the ρ(*T*) dependence in zero magnetic field (Fig. 1b). To probe any possible electronic nematicity inherent to AFQ order the transverse magnetoresistance was measured for the CeB_6_ single crystal by applying of dc current J along^1–10^ axis and magnetic field B along [001], [111] or [110] crystallographic directions (Fig. 1e). Selected experimental data are shown in Fig. 1c and Fig. 1d for 1.85 K and 4.2 K, respectively. Application of a magnetic field reduces significantly the resistivity, which decreases by a factor of 22 in 8 T at the lowest available temperature 1.85 K (Fig. 1c). This negative magnetoresistive effect dominates in AFQ and P phases (Fig. 1c,d). However, while magnetoresistance of CeB_6_ is independent on the magnetic field direction in the P phase, a pronounced anisotropy of resistivity appears in AFQ phase (Fig. 1d). The comparison of ρ_i_/ρ_j_ ratios, where the indices i, j = {100; 110; 111} point to magnetic field direction, shows that both ρ_110_/ρ_100_ and ρ_111_/ρ_100_ demonstrate similar complicated behavior in AFQ phase while ρ_110_/ρ_111_ deviates from 1 not more than by 1-2% (see Fig. 1c,d). On the contrary, the ρ_110_/ρ_100_ (and equivalently ρ_111_/ρ_100_) ratio changes the amplitude from ρ_110_/ρ_100_ < 1 to ρ_110_/ρ_100_ > 1 reaching the value 1.1 at 1.85 K. This observation proves definitely that the [100] axis in CeB_6_ should be considered as the selected one in the expected “electron liquid crystal” nematic phase. This conclusion is in full agreement with the ESR data, where [100] axis corresponds to the strongest spin fluctuations at low temperatures^8^. At the same time, the origin of oscillations in ρ_110_/ρ_100_ and ρ_111_/ρ_100_ for *B* > 3–4 T, which amplitude noticeably exceeds experimental errors (Fig. 1d), will be discussed elsewhere.

Assuming inequivalence of [100] and [110]/[111] directions axis, the **J||** [001] geometry was chosen to study the angular dependences of the resistivity ρ(*B*, *T*, *θ*) in cubic single crystals of CeB_6_ (Fig. 1f). Thus a dc current is applied along the 4-fold axis of symmetry so that magnetic field **B** applied perpendicular to **J** passes through [100] and [110] axis. Hereafter the angle *θ* is measured from [110].

In the considered case, if the symmetry of the magnetic scattering or spin fluctuations is related to the crystal symmetry solely and all Ce^3+^ ions may be treated as equivalent, it is possible to expect either isotropic magnetoresistance for symmetric cubic structure, or dominating 4-fould axis if any anisotropy of ρ(*B*, *T*, *θ*) exists. The crystal symmetry is the same in the P and AFQ phases, so if the orbital ordering effects are not involved one can expect the same type of the angular dependence in both paramagnetic and antiferroquadrupole phases. When the orbital degree of freedom is taken into account, the chess board type of quadrupole moments ordering in the AFQ phase (Fig. 1f) will lead to dominating *P*_2_(cos*θ*) in the ρ(*B*, *T*, *θ*) if any anisotropy develops^6^. In the P phase, the orbitals of Ce^3+^ with different quadrupolar charges are disordered and the angular dependence should be smeared.

It is worth noting that any precise measurements of magnetoresistance angular dependences in CeB_6_ are problematic as long as resistivity in the AFQ phase strongly depends on temperature (Fig. 1c,d). This requires high stability of temperature and magnetic field during the angular scans (see Methods section and ref.^18^ for more details). Successful solution of the mentioned experimental problems allowed obtaining accurate ρ(*θ*) data.

**Figure 2:**
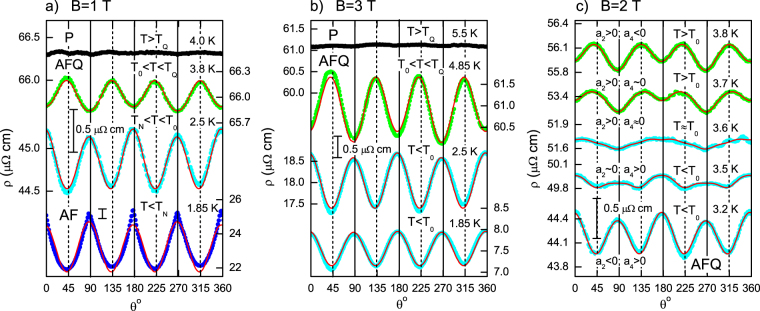
Selected angular dependences of the resistivity ρ(*θ*, *B*_0_, *T*_0_) in fixed magnetic fields *B*_0_ = 1 T (**a**) and *B*_0_ = 3 T (**b**) at various temperatures for the **J||** [001] geometry (Fig. 1f). Solid circles present the experimental data, red lines are the fits calculated with the help of Eq. 3 accounting *P*_2_(cos*θ*) and *P*_4_(cos*θ*) terms. The evolution of the ρ(*B*, *T*, *θ*) angular dependences near the sign inversion points of *a*_2_ and *a*_4_ for *B*_0_ = 2 T is shown in panel c. Vertical bars in all windows correspond to the same value of 0.5 μΩ·cm. Note the different scale for ρ(*B*, *T*, *θ*) data in the AF phase (panel a). Vertical solid and dashed lines correspond to the families of <110> and <100>, respectively.

Selected angular dependences of the resistivity ρ(*B* = const, *T*, *θ*) measured at different temperatures in magnetic fields 1 T, 3 T and 2 T are shown in Fig. 2 (panels a, b and c respectively). At first we shall analyze data for 1 T and 3 T. In the P phase (*T* > *T*_*Q*_(*B*)) the resistivity is isotropic within experimental accuracy. Entering into the AFQ phase results in development of the angular modulation of magnetoresistance, which amplitude increases with lowering temperature (Fig. 2). It is remarkable that the sign of the contribution from the angular oscillations to total resistivity depends on temperature. Above a certain temperature *T*_0_ the axes [100] and [110] correspond to the minimum and maximum of the ρ(*θ*) respectively, whereas for *T* < *T*_0_ the situation changes to opposite, so that minima of the resistivity match the [100] directions (Fig. 2a,b).

In order to parametrize the observed angular dependences we performed analysis of experimental data assuming

3 $$\rho (B,T,\theta )={\rho }_{0}(B,T)\cdot f(\theta ),$$

where ${\rho }_{0}(B,T)=\rho (B,T,\theta =0)$ denotes the resistivity value for ***B***||[110] direction, and *f*(*θ*) is given by Equation (1). As a first step, the coefficients *a*_*n*_ were bound by standard expansion procedure into Legendre polynomial series^5^. The results obtained may be summarized as follows. (1) The P phase is characterized by *a*_*n*_, which are two or three orders of magnitude less than those in the AFQ or AF phase. (2) The odd coefficients in the AFQ or AF phases are also at least two orders of magnitude less than even ones. (3) For *B* ≤ 3 T the contribution of the second and fourth Legendre polynomials in the AFQ phase dominates and *f*(*θ*) is given by the first two terms in Equation (2). (4) In magnetic fields *B* > 3 T higher even harmonica contribute essentially to the angular dependences of resistivity in the AFQ phase. For that reason, we will confine ourselves here by results obtained in the low field range *B* ≤ 3 T and the data corresponding to higher magnetic fields will be a subject of separate publication.

**Figure 3:**
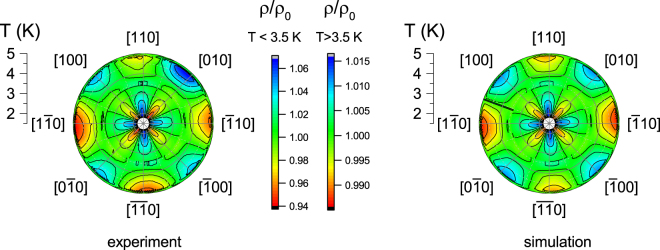
Representation of the reduced angular dependences of the resistivity ρ(*B*, *T*, *θ*)/ρ_0_ in polar graphs for magnetic field 3 T. Panel a displays experimental data. Panel b shows the result of simulation assuming two terms expansion of *f*(*θ*) (*P*_2_(cos*θ*) and *P*_4_(cos*θ*)). Note different scales of ρ/ρ_0_ for *T* < *T*_0_ ≈ 3.5 K and *T* > *T*_0_ ≈ 3.5 K.

The coefficients *a*_2_ and *a*_4_ may be also found by direct two-parameter fitting of the experimental ρ(*θ*) curves with the help of Equations (2–3). In the AFQ phase, the difference provided by both methods never exceeds ~2%. Solid lines in Fig. 2a,c show that $f(\theta )=1+{a}_{2}{P}_{2}(\cos \,\theta )+{a}_{4}{P}_{4}(\cos \,\theta )$ fits to the experimental data in the AFQ phase very well. In contrary, the experimental curve in the AF phase becomes distorted with respect to this model approximation (Fig. 2a). The correctness of the procedure suggested for data analysis in the AFQ phase is also confirmed by comparison of the experimental ρ(*B*, *T*, *θ*) data and those ones simulated by two coefficients *a*_2_ and *a*_4_. The respective colored maps in polar plot (Fig. 3) demonstrate a remarkable agreement between the experimental and simulated data. Thus the “liquid crystal" equations (1–2) with only few terms of expansion are applicable for description of experimental data in the single crystal of CeB_6_.

**Figure 4:**
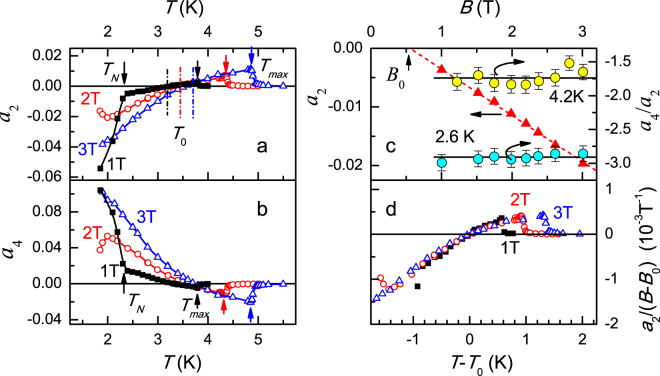
Parameters *a*_2_ and *a*_4_ of decomposition by Legendre polynomials of the magnetoresistance angular dependences in fixed magnetic fields 1, 2 and 3 T, as functions of temperature (panels a,b). Characteristic temperatures *T*_*max*_(*B*) and *T*_*N*_ are marked by arrows, the temperatures *T*_0_(*B*) are denoted in the panel a by dashed lines. Panel c shows field dependences of *a*_2_ at *T* = 2.6 K and those of ratio *a*_4_/*a*_2_ for *T* > *T*_0_ (*T* = 4.2 K) and *T* < *T*_0_ (*T* = 2.6 K). Panel d display scaling relation for *a*_2_ coefficient (see text for details).

Figure 3 shows also a change of the magnetic scattering symmetry under lowering the temperature below *T*_0_ that leads to rotating of the magnetoresistance angular dependence pattern by 45 degrees. This feature is a consequence of the *a*_2_(*T*) and *a*_4_(*T*) temperature dependences observed in fixed magnetic fields (Fig. 4). An abrupt onset of the magnetoresistance anisotropy occurs at characteristic temperature *T*_*max*_(*B*), which corresponds to a stepwise increase in the absolute values of *a*_2_ and *a*_4_. Note that *T*_*max*_(*B*) coincides practically with the phase boundary for P → AFQ transition (stars in Fig. 1a).

At temperatures close to *T*_*max*_(*B*), the coefficients *a*_2_ and *a*_4_ are positive and negative, respectively (Fig. 4). When temperature is lowered, the *a*_2_ parameter decreases and changes sign at some particular temperature *T*_0_(*B*) (dashed lines in Fig. 4a). Simultaneously the coefficient *a*_4_ increases and changes sign also in the vicinity of *T*_0_(*B*), but not exactly at the same temperature as *a*_2_ (Fig. 4b). This temperature evolution of the coefficients *a*_2_ and *a*_4_ means that the symmetry of spin fluctuations, scattering on which control magnetoresistance in CeB_6_, changes around *T*_0_(*B*) (see, e.g., data in Fig. 2c) so that the ρ(*B*, *T*, *θ*) pattern rotates at 45 degrees (Fig. 3). Due to the negative magnetoresistance in CeB_6_ we expect that for *T* > *T*_0_(*B*) the directions of maximal spin fluctuations is [110], whereas in the range *T* < *T*_0_(*B*) the strongest spin fluctuations occur along [100] (Figs 2–3). It is worth noting that ESR in the AFQ phase was observed for *T* < *T*_0_(*B*) exceptionally^8^ and the corresponding evidence for the enhancement of the spin fluctuations along [100] axis does not contradict to the expected change of the spin fluctuations symmetry, which follows from the results of our work.

Important direct information about the spin fluctuations symmetry can be extracted from the zoomed view of the ρ(*B*, *T*, *θ*) data in the vicinity of *T*_0_ presented for *B* = 2 T in Fig. 2c. It is clearly seen that lowering of temperature results in the continuous increase of *a*_4_ parameter so that an exotic situation is realized at temperatures corresponding to its zero value. Indeed, a distinct *P*_2_(cos*θ*) picture is detected for the 4-fold axis of symmetry (see 3.6 K data in Fig. 3c). The established lowering of symmetry reveals a nonequivalence of two <110> directions (at *θ* = 0°/180° and *θ* = 90°/270°) and appears to prove definitely the existence of spatial anisotropy in this cubic system. At the same time, no difference in the resistivity values is observed for the family of B|| <100> directions (*θ* = 45°, 135°, 225° and 315°, Fig. 2c).

In the studied case, the rotation around [001] direction corresponds to the 4-fold axis, if orbital ordering effects are not important. This is true for the P phase, where magnetoresitance is isotropic, so that this type of symmetry does not produce any anisotropy itself. The reduction of the symmetry from 4-fold to 2-fold axis giving rise to *P*_2_ term is a consequence of entering into the AFQ phase^6^ with the chess board type ordering of the quadrupolar moments (Fig. 1f). However, the experimental situation is more complicated because both *P*_2_ and *P*_4_ terms are observed simultaneously. Therefore the relation of the experimental magnetoresistance data to the crystal symmetry is not so straightforward in CeB_6_ and, in our opinion, could not be foreseen a priori, even in view of the existing theory of nematic effects in the quadrupolar systems^6^.

Entering into AF phase induces the abrupt increase of the *a*_2_(*T*) and *a*_4_(*T*) absolute values (see data for 1 T in Fig. 4a,b). As long as AFQ phase is characterized by the field induced antiferromagnetism, this observation indicates a possible link between AF ordering in CeB_6_ and development of the magnetoresistance anisotropy, which is caused by either temperature or magnetic field. Therefore it is useful to consider the *a*_2_ and *a*_4_ field dependences at various temperatures. As long as the observation of the *P*_4_ and *P*_2_ terms together is unusual and unexpected in theory it is interesting to analyze a possible link between them. The qualitative similarity of the *a*_2_(*T*) and *a*_4_(*T*) behavior (Fig. 4a,b) suggests analyzing the ratio *b* = *a*_4_/*a*_2_. Figure 4c shows that parameter *b* does not depend on magnetic field up to 3 T within experimental accuracy, but its value is different above and below *T*_0_. In the range *T* > *T*_0_ the ratio *b* = *a*_4_/*a*_2_ is about −1.75, while for *T* < *T*_0_ this parameter equals approximately −2.9 (see data for 4.2 K and 2.6 K, inset in Fig. 4). This finding proves definitely that the *P*_2_ and *P*_4_ contributions in CeB_6_ are closely interrelated and the analytical approximation of resistivity in the AFQ phase of CeB_6_ for *B* < 3 T may be given by

4 $$\rho (B,T,\theta )={\rho }_{0}(B,T)\cdot f(\theta )=\rho (B,T,0)\cdot [1+{a}_{2}(B,T)\cdot ({P}_{2}(\cos \,\theta )+b\cdot {P}_{4}(\cos \,\theta ))],$$

where the essential temperature and field dependence of *f*(*θ*) is due to *a*_2_(*B*, *T*) and the coefficient *b* undergoes stepwise variation at *T*_0_(*B*). Thus we can conclude that *a*_2_(*B*, *T*) is the principal parameter, which is responsible for the unusual anisotropy of magnetoresistance in CeB_6_. In this respect, it is reasonable to relate this parameter with the orbital ordering process in the AFQ phase developing in cerium hexaboride at low temperatures.

Consideration of the field dependence of *a*_2_ shows that this parameter increases almost linearly with the magnetic field (panel c in Fig. 4). The linear trend suggests that *a*_2_(*B*) varies as *a*_2_~(*B*-*B*_0_) with *B*_0_~0.3-0.5 T. Hence, either the threshold magnetic *B*_0_ field for nematic effect may be expected, or, at least, the type of field dependence is changed in the range of low magnetic fields. Apparently, it is difficult to resolve this issue experimentally, as long as magnetoresistance magnitude decreases with magnetic field. For that reason, a scaling analysis is performed in this work to elucidate the character of the field and temperature dependence of *a*_2_. We found that *a*_2_(*B*, *T*) may be expressed as

5 $${a}_{2}=(B-{B}_{0})\cdot {\phi }(T-{T}_{0}(B)).$$

Here *φ* (*T*) denotes universal scaling function of temperature plotted in Fig. 4d. As a remarkable feature of the observed scaling it is possible to mark that Equation (5) is valid not only for *T* < *T*_0_, but as well in the range *T* > *T*_0_ and scaling relation lasts up to *T*_*max*_(*B*) (Fig. 4d). Considered scaling is not limited by the case of the AFQ phase, but it also holds in the AF phase. It is also amazing that *a*_2_(*B*, *T*) does not scale with *T*_*max*_(*B*) = *T*_*Q*_(*B*), i.e. with the P → AFQ transition temperature. In addition, the validity of Equation (5) supports the possible threshold behavior of the magnetoresistance angular dependences.

An unambiguous establishing of the electron nematic effect in CeB_6_ requires consideration of any other possible mechanisms, which may either emulate 2-fold symmetry or fade it in a real experimental situation. For example, a misalignment of the sample will cause rotation of the sample around axis tilted by some random angle φ with respect to [001]. This will result in elliptically modulated magnetic field in the plane perpendicular to [001] and appearance of additional even contribution, which originates from projection of the magnetic field on [001] axis. In principle, this effect can induce the *P*_2_ term as in electron nematic effect, although the orientation of the director in this case will not be fixed with respect to crystallographic directions due to the random character of the misalignment. From experimental data for the field dependences of resistivity along different crystallographic directions (Fig. 1) we have estimated the magnitude of the relative distortion δρ/ρ of the ρ(θ) curve caused by the considered effect. For the case of AFQ phase and *B* = 3 T, calculation gives δρ/ρ~3·10^−4^ for misalignment with φ = 1° corresponding to the expected accuracy (see Methods section) and δρ/ρ~3 · 10^−3^ for the unlikely big error in the sample orientation φ = 5°. Such small distortions unable explaining observed magnitudes of the modulation of the resistivity angular dependences in the AFQ phase (Fig. 2). Simultaneously, our calculations show that the misalignment caused distortion in our experiments may become somewhat bigger in the AF phase and, in principle, may be partially responsible for the observed departures of our ρ(*θ*) dependences from the equations (2–3) (Fig. 2a). However, even in this case this effect does not allow any quantitative interpretation of the experimental ρ(*θ*) data.

In some electronic systems, the nematic effects may be masked by the formation of the domain structure, which occurs, e.g., in the case of ‘122’ Fe-arsenide superconductors^14^. However, this is not necessary the case for the highly symmetric systems with quadrupolar order. If the domain structure is assumed to exist in CeB_6_, it is necessary to postulate the presence of domains with different quadrupolar order as long as nematic effect develops just in the AFQ phase. So far there is neither clear experimental nor explicit theoretical evidence that such domains exist in this phase. The currently accepted view is that the AFQ phase is homogeneous on the macroscopic scale. Nevertheless, the quasi-threshold character of *a*_2_(*B*) field dependence (Fig. 4) may be hypothetically attributed to lifting of the domain degeneracy by application of the magnetic field similar to the case of ‘122’ Fe-arsenide superconductors^14^. However, in the case of CeB_6_, the reflex with **k** = [1/2, 1/2, 1/2] related to the orbital ordering may be observed in zero magnetic field in the single crystals^19^ so the hypothetical smearing due to domain structure should be somehow broken even at6 *B* = 0^19^. Therefore currently there are no unambiguous reasons to involve domains into the problem of nematic effects in the quadrupolar system CeB_6_, although further studies may shed more light on this intriguing question.

In the present study, we repeated our experiments on several single crystals and measured the angular dependences after several mountings of the same crystal into the experimental setup. Nice agreement of all the data obtained allowed us to conclude that in our case the reproducibility of the results is not related to any misalignment (which produces a small effect on ρ(θ) curves) and is due to the intrinsic properties of this homogeneous AFQ phase.

The above analysis confirms the interpretation of the *P*_2_ term in the angular dependence of the magnetoresistance in the AFQ phase of CeB_6_ as caused by the electron nematic effect. The set of experimental data including qualitative changes in the symmetry of magnetic scattering (Figs 2, 3), the change of the *b* = *a*_4_/*a*_2_ ratio, validity of the scaling equation (5) and onset of the electron spin resonance strongly supports the idea that *T*_0_(*B*) is a kind of the new transition located inside the AFQ phase, which is well separated from *T*_Q_(*B*) (Fig. 1a). Simultaneously, the expected threshold behavior indicates that characteristic field *B*_0_ may be related to some transition occurring in weak magnetic field between AF and AFQ phases (Fig. 1a). It is worth noting that such line located on the magnetic phase diagram close to estimated *B*_0_ value was earlier predicted theoretically^20^ and observed experimentally^21^.

We wish to point out, that the most of the theoretical studies of the orbital ordering in CeB_6_ treat the AFQ phase as magnetically homogeneous one and no extra boundaries like *T*_0_(*B*) are expected. More work is required to elucidate the microscopic nature of *T*_0_(*B*) and possible influence of the change of spin fluctuations regime on various physical properties. We would like to add that straightforward association of the angular dependences of the resistivity with the chess board type alignment of the Γ_8_ wave functions quadrupolar moments in the AFQ phase do not favor simultaneous appearance of the *P*_2_(cos*θ*) and *P*_4_(cos*θ*) terms in expansion of the experimental dependences ρ(*B*, *T*, *θ*). The aforementioned difficulties require further development of existing theory in order to reach full interpretation of the results of the present work.

The analytical form derived from the analysis of experimental data (see Eq.(4)) indicates that, in analogy with liquid crystals, the *a*_2_(*B*, *T*) coefficient may be treated as an order parameter for electron nematic effect, which develops in the orbitally ordered AFQ phase of CeB_6_. However, the observed nematic effect is not simple and reorientation of the director axis at some temperature takes place. The behavior of the order parameter for this transition, especially its temperature variation, is rather unusual and does not meet the case of the standard Landau theory. The change of sign at *T*_0_ and presence of two transition temperatures *T*_0_ and *T*_*max*_ = *T*_*Q*_ indicates possible complex nature of *a*_2_(*B*, *T*) due to a combination of several positive and negative contributions. Analysis of this possibility also requires development of the adequate theory, which is missing to date.

Summarizing up, the detailed study of the angular dependences of the magnetoresistance in the antiferroquadrupole phase of CeB_6_ revealed the electron nematic effect developing when magnetic field exceeds some characteristic value ~0.3-0.5 T. New transition inside the AFQ phase, which may be associated with the change of the symmetry of magnetic scattering on spin fluctuations, is discovered.

## Methods

High quality single crystals of CeB_6_ identical to those studied earlier in^8^ were investigated. The initial ingots of single crystals of CeB_6_ were oriented with the help of X-rays. After cutting the sample, the resulting orientation was checked again by X-ray diffraction and, if necessary, the sample shape was corrected by additional cutting or polishing to get desirable accuracy of the crystal axes alignment with respect to the sample faces and edges. Application and repeating of this procedure resulted in orientation error less than 1°. Mounting of the sample into the experimental setup, which allowed precise sample rotation and positioning in a magnetic field, was done under optical microscope control without adding additional errors in the sample alignment.

The angular dependences of the magnetoresistance were measured with the help of the experimental setup, which allows 360° rotation of the sample by discrete steps of 1.8° in magnetic field *B* up to 8 T supplied by superconducting magnet. The accuracy of the sample temperature stabilization was about 2 mK in the range *T* < 10 K. Recent example of the application of this setup for studying of magnetoresistance anisotropy in strongly correlated materials may be found elsewhere^18^.

Single crystals of CeB_6_ were cut in a way that the dc current **J** is applied along the^1–10^ or [001] directions. As a result, magnetic field **B**, which is transverse to **J**, passes through the principal crystallographic axes when the sample is rotated around **J** direction (Fig. 1e,f).
